# Supplementary material for: Cytotoxic Effects of Major and Emerging Mycotoxins on HepaRG Cells and Transcriptomic Response after Exposure of Spheroids to Enniatins B and B1
Source: Toxins (Basel). 2024 Jan 18;16(1):54. doi: 10.3390/toxins16010054 (PMC10819306; doi:10.3390/toxins16010054)
Supplement: Supplementary file 1 [file toxins-16-00054-s001.zip › toxins-2778243-supplementary.pdf]

Supplementary Materials: Cytotoxic Effects of Major and Emerging Mycotoxins on HepaRG Cells and Transcriptomic Response after Exposure of Spheroids to Enniatins B and B1

France Coulet, Monika Coton, Cristian Iperi, Marine Belinguer Podevin, Emmanuel Coton and Nolwenn Hymery

Table S1: Statistic alignments of Sample Control and ENN B/B1 treatments.

Sample Name	Total Raw Reads	Total HQ Reads	HQ Bases (Q30)	GC Content	Mean Read Length (bp)	HQ Reads %
**ENNB_D_**IC_{30}	67.9 M	67.07 M	91,0%	49,0%	150	98,8%
**ENNB_UN_**IC_{30}	78.21 M	77.11 M	90,8%	48,8%	150	98,6%
**ENNB1_D_**IC_{0}	87.8 M	86.35 M	90,0%	49,0%	150	98,4%
**ENNB1_UN_**IC_{0}	67.24 M	66.59 M	91,8%	48,9%	149	99,0%
**ENNB1_D_**IC_{10}	76.88 M	75.92 M	90,9%	49,1%	150	98,8%
**ENNB1_UN_**IC_{10}	72.75 M	72.04 M	91,8%	48,9%	149	99,0%
**ENNB1_D_**IC_{30}	74.21 M	73.66 M	94,4%	49,8%	150	99,3%
**ENNB1_UN_**IC_{30}	60.05 M	59.3 M	90,1%	49,0%	149	98,7%
**Control_D**	60.62 M	59.95 M	91,5%	48,5%	150	98,9%
**Control_UN**	65.82 M	65.12 M	91,2%	48,8%	149	98,9%

Table S2: Tested mycotoxin concentrations in the different *in vitro* cell culture models. All concentrations correspond to the final mycotoxin doses added to the media, dilution included.

Mycotoxins	Molar weight	Initial concentration (mol/L)	Concentration range (mol/L)
DON	296.32	3.37×10^{-3} (in DMSO)	30×10^{-6}; 24×10^{-6}; 20×10^{-6}; 16×10^{-6}; 12×10^{-6} ; 8.10^{-6}; 4.10^{-6}; 2.10^{-6}; 1.10^{-6} ; 5×10^{-7}
T-2	466.52	21.44×10^{-3} (in DMSO)	30×10^{-6}; 10×10^{-6}; 5×10^{-6}; 1×10^{-6}; 5×10^{-7}; 1×10^{-7}; 5.10^{-8}; 1.10^{-8}
ZEA	318.36	6.28×10^{-3} (in DMSO)	12.56×10^{-5}; 90×10^{-6}; 75×10^{-6}; 60×10^{-6}; 45×10^{-6}; 30×10^{-6}; 15×10^{-6}
ENN B	639.32	3.13×10^{-3} (in water)	41.8×10^{-6}; 20.9×10^{-6}; 10.4×10^{-6}; 1.04×10^{-6}; 1.04×10^{-7}; 1.04×10^{-8}; 1.04×10^{-9}
ENN B1	653.85	3.06×10^{-3} (in water)	40.8×10^{-6}; 20.4×10^{-6}; 10.2×10^{-6}; 1.02×10^{-6}; 1.02×10^{-7}; 1.02×10^{-8}; 1.02×10^{-9}
ENN A	681.9	2.93×10^{-3} (in water)	39×10^{-6}; 19.5×10^{-6}; 9.76×10^{-6}; 9.76×10^{-7}; 9.76×10^{-8} ; 9.76×10^{-9}; 9.7×10^{-10}
ENN A1	667.87	0.74×10^{-3} (in water)	10×10^{-6}; 5×10^{-6}; 2.5×10^{-6}; 2.5×10^{-7}; 2.5×10^{-8} ; 2.5×10^{-9}; 2.5×10^{-10}
BEA	783.95	2.55×10^{-3} (in water)	34.10^{-6}; 17.10^{-6}; $8.5.10^{-6}$; $8.5.10^{-7}$; $8.5.10^{-8}$; $8.5.10^{-9}$

Table S3: Inhibitor concentration (“IC_0”, “IC_{10}” and “IC_{30}”) used to treat spheroids for RNA-seq analysis.

**Inhibitory concentration**	**Mycotoxins**	
	Enniatin B1	Enniatin B
**Negative control**	0	0
IC_0 **(µM)**	0.1	/
IC_{10} **(µM)**	1.37	/
IC_{30} **(µM)**	1.96	520

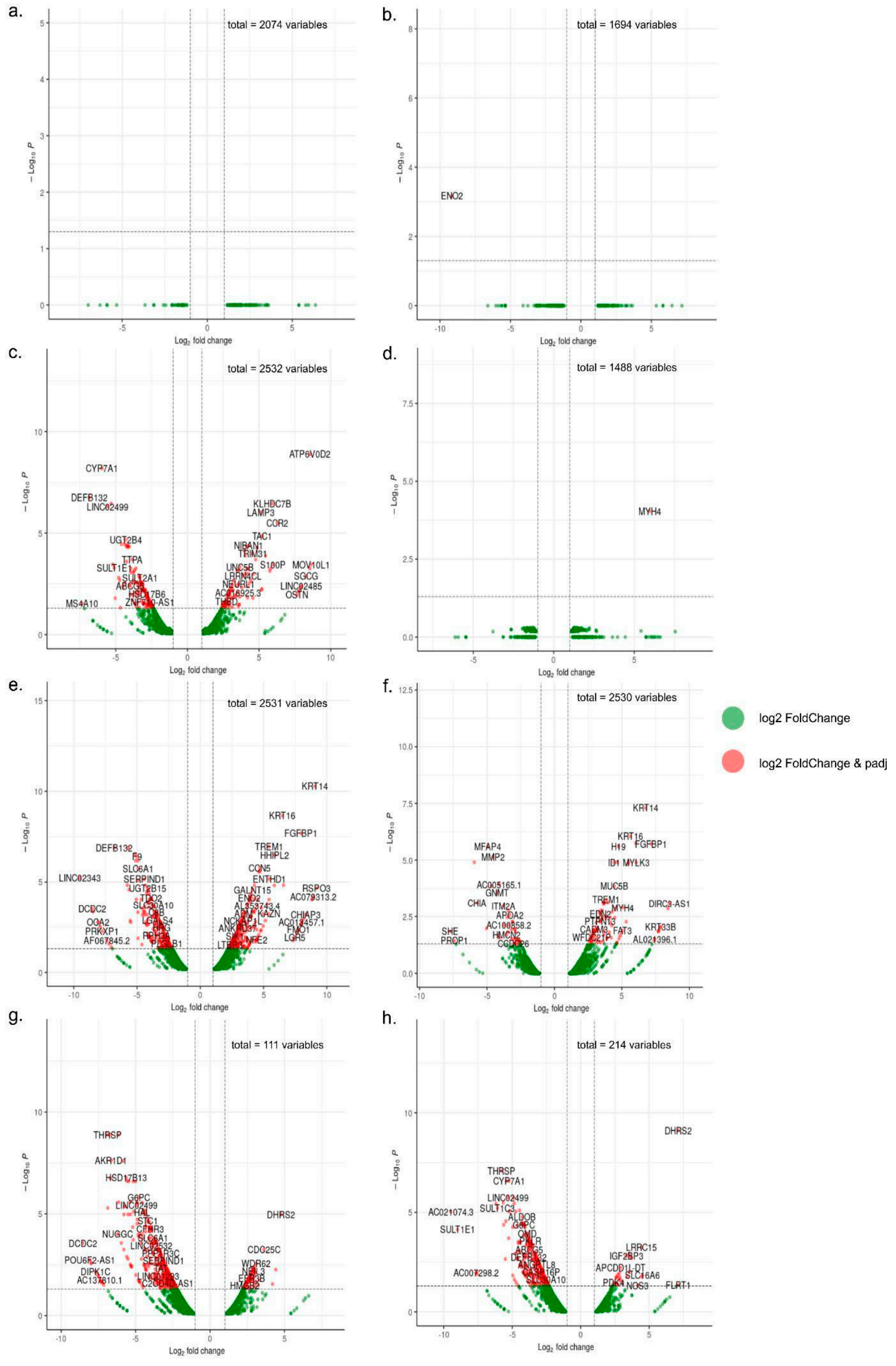

Figure S1: Volcano plot from spheroid RNA extraction. X-axis represents log2FodChange and the y-axis represents the p-value adjusted. (a.) ENN B1/ IC_0 on differentiated spheroid. (b.) ENN B1/ IC_0 on undifferentiated spheroid. (c.) ENN B1/ IC_{10} on differentiated spheroid. (d.) ENN B1/ IC_{10} on undifferentiated spheroid. (e.) ENN B1/ IC_{30} on differentiated spheroid. (f.) ENN B1/ IC_{10} on undifferentiated spheroid. (g.) ENN B/ IC_{30} on differentiated spheroid. (h.) ENN B/ IC_{30} on undifferentiated spheroid.
